# Characterization of novel endo-β-*N*-acetylglucosaminidases from *Sphingobacterium* species, *Beauveria bassiana* and *Cordyceps militaris* that specifically hydrolyze fucose-containing oligosaccharides and human IgG

**DOI:** 10.1038/s41598-017-17467-y

**Published:** 2018-01-10

**Authors:** Yibo Huang, Yujiro Higuchi, Takashi Kinoshita, Ai Mitani, Yasunari Eshima, Kaoru Takegawa

**Affiliations:** 1 Department of Bioscience and Biotechnology, Faculty of Agriculture, Kyushu University, 6-10-1 Hakozaki, Fukuoka, 812-8581 Japan; 2 Fushimi Pharmaceutical Co. Ltd., Marugame, Kagawa, 763-8605 Japan

**Keywords:** Hydrolases, Glycobiology

## Abstract

Endo-β-*N*-acetylglucosaminidase (ENGase) catalyzes hydrolysis of *N*-linked oligosaccharides. Although many ENGases have been characterized from various organisms, so far no fucose-containing oligosaccharides-specific ENGase has been identified in any organism. Here, we screened soil samples, using dansyl chloride (Dns)-labeled sialylglycan (Dns-SG) as a substrate, and discovered a strain that exhibits ENGase activity in the culture supernatant; this strain, named here as strain HMA12, was identified as a *Sphingobacterium* species by 16S ribosomal RNA gene analysis. By draft genome sequencing, five candidate ENGase encoding genes were identified in the genome of this strain. Among them, a recombinant protein purified from *Escherichia coli* expressing the candidate gene ORF1188 exhibited fucose-containing oligosaccharides-specific ENGase activity. The ENGase exhibited optimum activities at very acidic pHs (between pH 2.3–2.5). A BLAST search using the sequence of ORF1188 identified two fungal homologs, one in *Beauveria bassiana* and the other in *Cordyceps militaris*. Recombinant ORF1188, *Beauveria* and *Cordyceps* ENGases released the fucose-containing oligosaccharides residues from rituximab (immunoglobulin G) but not the high-mannose-containing oligosaccharides residues from RNase B, a result that not only confirmed the substrate specificity of these novel ENGases but also suggested that natural glycoproteins could be their substrates.

## Introduction

Endo-β-*N*-acetylglucosaminidases (ENGases, EC 3.2.1.96) are a class of enzymes, which can hydrolytically cleave β-1,4 glycosidic bonds within the *N*,*N*′-diacetylchitobiose moiety in the inner-core region of *N*-glycan and release glycan chain from their associated proteins, thus leaving one unit of *N*-acetylglucosamine (GlcNAc), with or without the core fucose, linked to the asparagine residue^1^. ENGases are widely distributed in various organisms, including bacteria, fungi, plants, animals and humans. According to the Carbohydrate-Active Enzymes (CAZy) database (http://www.cazy.org/)^2^, ENGases are classified based on their amino acid sequence homologies into one of two glycoside hydrolase (GH) families, either family GH18 or family GH85. Normally, GH18 endoglycosidases only exhibit hydrolytic activity; members of this family of enzymes include Endo-H from *Streptomyces plicatus*^3^, Endo-F1, Endo-F2, and Endo-F3 from *Elizabethkingia meningoseptica*^4^, Endo-S and Endo-S2 from *Streptococcus pyogenes*^5^, Endo-T from *Tricoderma reesei*^6^, Endo-FV from *Flammulina velutipes*^7^, and Endo-Sd from *Streptococcus dysgalactiae* subspecies *dysgalactiae*^8^. In contrast, GH85 endoglycosidases, which include Endo-A from *Arthrobacter protophormiae*^9^, Endo-M from *Mucor hiemalis*^10^, Endo-D from *Streptococcus pneumoniae*^11^, Endo-CE from *Caenorhabditis elegans*^12^, Endo-BH from *Bacillus halodurans* C-125^13^, Endo-Om from *Ogataea minuta*^14^, and Endo-CC1 and Endo-CC2 from *Coprinopsis cinerea*^15^, exhibit both hydrolytic and transglycosylation activities, enabling them to be used for broad applications for glycosylation remodeling of heterogeneous glycopeptides, glycoproteins and glycoconjugates.

In this study, we screened soil samples for microorganisms exhibiting ENGase activity. We identified four candidate ENGases in *Sphingobacterium* species, encoded by ORF1152, ORF1188, ORF3046 and ORF3750, collectively called here as Endo-SBs, and characterized the enzymatic properties of proteins expressed by these genes in detail. We found two homologs of ORF1188 in fungi *Beauveria bassiana* and *Cordyceps militaris* and cloned them. Recombinant proteins, purified from *E. coli* strains expressing the ORF1188, *Beauveria* and *Cordyceps* ENGase genes, catalyzed hydrolysis of fucose-containing complex type oligosaccharides.

## Materials and Methods

### Strain isolation and genome analysis

Bacterial strains used in this study were isolated from the soil samples collected in Fukuoka prefecture, Japan. All strains were cultured in LB medium (2.5% LB powder, Merck). ENGase activity in the bacterial culture supernatant was determined using dansyl chloride (Dns)-labeled sialylglycan (Neu_2_Gal_2_GlcNAc_2_Man_3_GlcNAc_2_-Asn-Dns; Dns-SG, Fushimi Pharmaceutical Co.) as the substrate. Genomic DNA was extracted from the strain HMA12 using a NucleoSpin® Tissue kit (TAKARA) according to the instructions provided by the manufacturer. 16S ribosomal RNA gene sequence was analyzed using universal primers. Whole-genome shotgun sequencing of the strain HMA12 was carried out using MiSeq (Illumina). Sequencing assembling was done using the program platanus 1.2.1. Gene annotation was performed using Glimmer 3.02b and BLAST 2.2.26.

### Preparation of recombinant ENGase proteins

*E. coli* strain BL21-CodonPlus (DE3) (Stratagene) and the expression vector pET32b (Novagen) were used for all recombinant DNA cloning experiments carried out in this study. For pre- and main cultures, *E. coli* cells were grown at 37 °C and 30 °C in media MMIA (1.25% triptone, 2.5% yeast extract, 0.85% NaCl, 0.4% glycerol, 20 mM Tris-HCl pH 7.2, 30 mg/L ampicillin) and LBA (2.5% LB powder, 30 mg/L ampicillin), respectively. Endo-CC1 from *Coprinopsis cinerea* was overexpressed and purified following a procedure established in our laboratory^15^.

To construct recombinant expression plasmids, each one of these six candidate ENGase genes, nucleotides 88–981 of ORF1188, (encoding amino acid residues 30–327 and lacking the signal peptide), nucleotides 61–1005 of ORF1152 (encoding amino acid residues 21–335 and lacking the signal peptide), ORF3046, ORF3750, nucleotides 55–945 of *Beauveria* ENGase gene (encoding amino acid residues 19–315 and lacking the signal peptide) and nucleotides 55–945 of *Cordyceps* ENGase gene (encoding amino acid residues 19–315 and lacking the signal peptide) was amplified by PCR from their respective genomic DNAs using the DNA polymerase PrimeStarGXL (Takara) and appropriate primers pairs (listed in Table S1). Each amplified DNA fragment was then ligated to a PCR amplified linear pET32b vector using the In-Fusion HD Cloning Kit (Takara) to create six expression plasmids. *E. coli* BL21(DE3) CodonPlus strain was transformed with each ENGase expression plasmid. Each transformed strain was precultured in MMIA medium at 37 °C overnight. Each preculture was inoculated into 250 ml of LBA liquid medium and culture OD_600_ was adjusted to 0.8–1.4. Next, 400 mM IPTG was added to each culture and cells were further grown overnight at 15 °C. Cells were pelleted by centrifugation at 7000 rpm for 7 min, each cell pellet was resuspended in 5 mL breaking buffer (300 mM NaCl, 200 mM Tris-HCl pH 7.5) and then lysed by ultrasonication on ice. Each cell lysate was centrifuged at 15000 rpm for 10 min at 4 °C to remove cell debris and the resulting supernatant was applied to a HisTrapTM FF 1 mL column (GE Healthcare). Recombinant protein purification was performed according to the manufacturer’s instructions. Resultant protein samples were concentrated by ultrafiltration using Amicon Ultra 0.5 ml filters (Millipore). Protein concentration was measured using the BCA Protein Assay Kit (Takara). The concentrated protein was subsequently used for the activity analysis.

### Analysis of hydrolase activity of ENGase proteins

The optimum pH for the hydrolase activity of each ENGase protein was assessed using pyridylamino (PA)-fucosyl sialobiantennary (Masuda Chemical) as a substrate and 100 mM acetate buffer; pH of the buffer was varied from pH 2.5 to pH 5.0 in increments of 0.5 pH unit using 100 mM acetate acid (pH 2.3). For this assay, since PA-sialobiantennary with terminal fucose was not commercially available, either 2 pmol of PA-sialobiantennary with core fucose (PA-fucosyl sialobiantennary) was mixed with a given amount of one of the newly identified *Sphingobacterium* ENGase recombinant protein (3.4 ng of ORF1188) or 2 pmol of PA-sialobiantennary was mixed either with 40 ng of recombinant *Beauveria* ENGase or with 77 ng of recombinant *Cordyceps* ENGase. The assay was carried out in 10 μL of 100 mM buffer (at an indicated pH) at 30 °C for 10 min or 20 min, following which the reaction was terminated by incubating the mixture at 99 °C for 10 min. The resultant samples were analyzed by HPLC (GL Science), which was equipped with a Wakosil 5C18 column (Wako), set at 40 °C. The HPLC was carried out using 50 mM ammonium acetate buffer (pH 4.0) containing 0.15% 1-butanol (Nacalai tesque) at a flow rate of 1.5 mL/min. Fluorescence emitted from PA (excitation at 320 nm, emission at 400 nm) was monitored, and the relative hydrolytic activity of ENGase proteins was determined from the peak area of hydrolyzed PA-fucosyl-acetylglucosamine.

To determine the substrate specificities of these ENGases, 2 pmol of each PA-labeled-oligosaccharide (Takara and Masuda Chemical) was mixed with a given amount of one of the recombinant ENGases (1.2 ng of ORF1188, 1 ng of *Beauveria* or 1.2 ng of *Cordyceps*) in 10 μL of 100 mM acetate buffer (pH 3) and the mixture was incubated at 30 °C for 20 min, following which the reaction was stopped by incubating the mixture at 99 °C for 10 min. The relative activity of each ENGase was determined in the same manner as was done above for determining the optimal hydrolytic activity by measuring the peak area of the hydrolyzed PA-fucosyl-acetylglucosamine or PA-acetylglucosamine.

To determine whether these recombinant ENGase proteins could hydrolyze glycoproteins, a given amount of each recombinant protein (5 μg of ORF1188; *Beauveria* and *Cordyceps* ENGases, 1 μg each) in 50 μL of 100 mM acetate buffer (pH 5.0) was incubated either with 10 μg of RNase B (Sigma) or with 10 μg of rituximab (Rituxan®; Zenyaku Kogyo Co., Ltd.) overnight at 30 °C. At the same time, 5 μg of Endo-CC1 in 50 μL of 100 mM phosphate buffer (pH 6.0) was incubated either with 10 μg of RNase B or with 10 μg of rituximab overnight at 37 °C. In addition, 200 unit of Endo-S (NEB) was incubated in parallel either with 10 μg of RNase B or with 10 μg of rituximab in 50 μL of 1x GlycoBuffer 1 overnight at 37 °C. Subsequently, all reaction samples were subjected to SDS-PAGE, following which gels were stained with Coomassie Brilliant Blue (CBB) EzStain AQua (Atto).

### LC-MS analysis

To analyze the oligosaccharide structure on rituximab, *Rapi*Fluor-MS labeling was performed according to the provider’s instructions (Waters). 10 μg of rituximab was diluted in a mixture of 3% *Rapi*Gest SF, and heated to 90 °C for 3 min, followed by cooling down to room temperature for 3 min. Rapid PNGase F was then added and the mixture was incubated for 5 min at 50 °C. To this mixture containing released glycans, 110 μL of *Rapi*Fluor-MS reagent was mixed and incubated at room temperature. Clean-up was carried out on a Glycoworks Hilic μElution plate conditioned first with water (200 μL) and then with 85% acetonitrile (ACN) in water (200 μL) before loading the samples. The wells were washed with 90% ACN and 1% formic acid in water (twice with 600 μL) and the labeled glycans were eluted with 30 μL of SPE elution buffer (three times). Finally, the samples were diluted with 100 μL of DMF and 210 μL of ACN. LC-MS experiments were performed on an Acquity H-Class Bio UPLC system equipped with UV and fluorescence, and Vion IMS Qtof, piloted by UNIFI 1.8.2 (Waters). *Rapi*Fluor-MS labeled *N*-glycans were analyzed on a Waters UPLC BEH Glycan column (130 Å, 1.7 μm, 2.1 × 150 mm) at 60 °C, using a gradient of 50 mM ammonium formate (pH 4.4) and 75–54% ACN over 35 min with a flow rate of 0.4 mL/min. Detection was achieved by monitoring fluorescence (265/425 nm) and carrying out mass spectrometry on m/z range 100–400 in the MS^E^ mode (Vion IMS Qtof; positive ion/sensitivity mode, capillary voltage: 2.75 kV, cone voltage: 80 V, source temperature: 120 °C, desolvation gas temperature: 600 °C, desolvation gas glow: 1200 L/h).

Intact MS-analysis was performed using samples of 20 μg rituximab that were incubated with or without 10 μg ENGases in 100 mM acetate buffer (pH 3.5) at 30 °C for 18 h. The LC-MS system was comprised of a Waters Acquity H-Class Bio UHPLC System with fluorescence and an MS detector. A Waters Vion IMS Qtof instrument was operated in positive ion/sensitivity mode, m/z range 400–4000. Capillary voltage was 2.75 kV, cone voltage was 140 V, source temperature was 150 °C and desolvation temperature was 600 °C. Instrument control, data processing, and deconvolution were performed using Waters UNIFI software v1.8.2. Samples were analyzed on a Waters ACQUITY UPLC BEH C4 column (300 Å, 1.7 μm, 2.1 × 50 mm) at 80 °C with a gradient of 0.1% formic acid in water and 0.1% formic acid in ACN (5%-55% of 0.1% formic acid in ACN for 5 min).

### Bioinformatic analysis

BLAST (http://blast.ncbi.nlm.nih.gov/Blast.cgi) searches were performed using nucleotide sequence of 16S rRNA gene or predicted protein sequences of ORF1152, ORF1188, ORF2117, ORF3046 and ORF3750 of *Sphingobacterium* species. Retrieved sequences were subjected to cluster analysis using the program CLUSTAL W (http://www.genome.jp/tools/clustalw/) and clustering was done using the neighbor-joining method. Domain search was carried out using the program Pfam (http://pfam.xfam.org/). Prediction of GH family was carried out using the program CAT (http://mothra.ornl.gov/cgi-bin/cat/cat.cgi).

### Accession numbers

Nucleotide sequences of ORF1152, ORF1188, ORF2117, ORF3046 and ORF3750 genes have been deposited in the DDBJ/EMBL/GenBank under the accession nos. LC306881, LC306882, LC306883, LC306884 and LC306885, respectively.

## Results

### Identification of a soil microorganism that exhibits ENGase activity

We isolated bacterial strains from over 100 soil samples to search for a fucose-containing oligosaccharide-specific ENGase. Culture supernatant of one isolated strain, here named as strain HMA12, exhibited ENGase activity using Dns-SG as a substrate (data not shown). To further identify this strain, we performed a BLAST search based on its 16S rRNA gene sequence, and found that it belongs to the *Sphingobacterium* species.

### Exploring for candidate ENGase genes in strain HMA12

**Figure 1:**
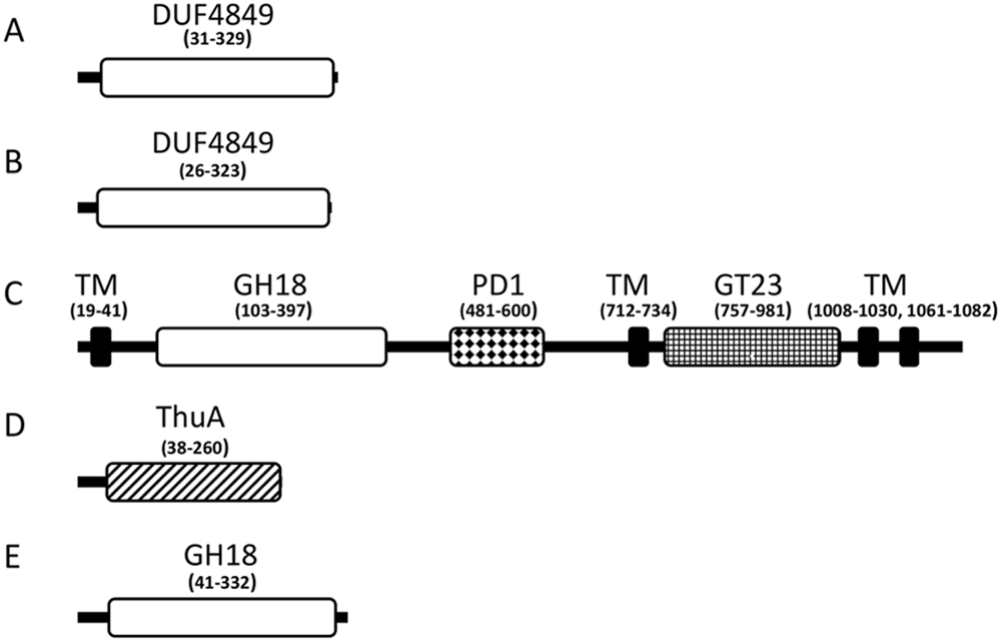
Domain structures of proteins encoded by ORF1152, ORF1188, ORF2117, ORF3046 and ORF3750 genes. The program Pfam was used to predict domains in each predicted ORF protein. Amino acid residue numbers corresponding to each domain are indicated in parenthesis. (**A**) ORF1152 containing DUF4849, which is a putative glycoside hydrolase family 18, chitinase_18 domain. (**B**) ORF1188 containing a DUF4849 domain. (**C**) ORF2117 containing four transmembrane (TM) domains (as predicted by SOSUI program), one glycosyl hydrolase family 18 (GH18) domain, one polysaccharide deacetylase (PD1) domain and on glycosyltransferase like family 2 (GT23) domain. **(D**) ORF3046 containing a trehalose utilisation A (ThuA) domain. (**E**) ORF3750 containing a GH18 domain.

To search for genes encoding ENGases, we performed whole-genome shotgun sequencing of strain HMA12. As a result, 4.70 Gbp was generated from 2.62 × 10^7^ sequencing reads with 727 fold-coverage, and 20 contigs were generated. Thus, we determined most of the genome sequence of HMA12, the details of which will be reported elsewhere. We next searched the genome sequence for ORFs exhibiting high sequence similarities to known ENGase genes and consequently found five candidate ORFs, named here as ORF1152, ORF1188, ORF2117, ORF3046 and ORF3750 (Table 1). Domain search analysis using the program Pfam predicted that the proteins encoded by ORF1152, ORF1188, ORF2117, ORF3046 and ORF3750 may have ENGase activity because they showed highest sequence similarities to reported ENGases (Fig. 1). Specifically, the ORF1152 and ORF1188 proteins harbored a putative glycoside hydrolase family 18 chitinase domain, which is known to have ENGase activity. The ORF2117, ORF3046 and ORF 3750 proteins, on the other hand, contained a GH18 family domain, which is typically seen in GH18 ENGases, based on analysis performed using the CAT program. Therefore, in order to obtain purified proteins for assaying enzyme activity, we next attempted to express these five candidate genes as recombinant proteins in *E. coli* cells. We were able to express 4 out of 5 of these ORFs (except ORF2117) in *E. coli*; this is probably because the protein encoded by ORF2117 is predicted to contain transmembrane domains. Thus, for further analysis we purified recombinant ORF1152, ORF1188, ORF3046 and ORF3750 proteins from *E. coli*.

**Table 1:** Candidate genes for endo-β-*N*-acetylglucosaminidases in strain *Sphingobacterium* species.

ORF	Homolog	Identity(%)	GH	Size(aa)
1152	Hypothetical protein [WP_052646382.1(*Sphingobacterium spiritivorum*)]	63	18	335
1188	Putative glycoside hydrolase Family 18, chitinase_18 [SEN99615.1 (bacterium A37T11)]	63	18	327
2117	Glycosyl transferase family 2 [WP_075993302.1(*Sphingobacterium* sp. B29)]	89	18	1140
3046	Glycosyl hydrolase [WP_050709498.1(*Dysgonomonas* sp. HGC4)]	71	18	263
3750	Glycoside hydrolase [OOG17107.1(*Sphingobacterium* sp. CZ-UAM)]	84	18	348

^a^Based on BLAST search using the amino acid sequences of the five ORFs, the corresponding homologs with the highest degree of identity are shown.

^b^Based on predictions by CAT program.

### Characterization of hydrolase activity of recombinant proteins

**Figure 2:**
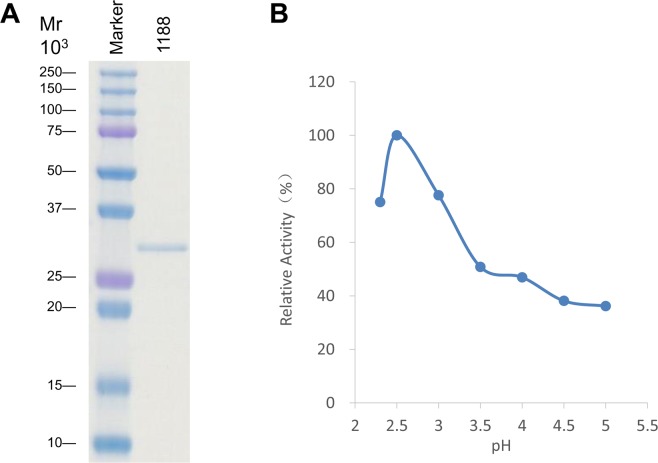
Analyses of molecular weight and pH-dependent activities of recombinant ORF1188 protein. (**A**) ORF1188 gene was expressed in *E. coli* as a recombinant protein and was purified. 0.5 μg of the purified protein sample was loaded onto a 15% acrylamide gel, and the gel was stained with CBB. (**B**) Effects of pH on the enzymatic activities of ORF1188 protein. Activity of the protein was assayed at various pHs using 100 mM acetate buffers.

To characterize the hydrolase activity of the putative Sphingobacterium ENGases, we individually expressed ORF1152 (without signal peptide), ORF1188 (without signal peptide), ORF3046 and ORF3750 in *E. coli*, and successfully purified these recombinant proteins from the respective *E. coli* cultures grown at 15 °C, as judged by SDS-PAGE analysis (Fig. 2A; images of original gels are shown in Fig. S1). Among these purified proteins, we found that only ORF1188 protein exhibited the ENGase activity. We further determined that the hydrolase activity of ORF1188 protein was optimum at pH 2.5 (Fig. 2B). The pH value for optimal activity is much lower than those observed for other ENGases.

Next, we characterized substrate specificities of ORF1188 protein. The relative activity of the protein was measured using varieties of PA-oligosaccharides as substrates, and the hydrolyzed products were analyzed by HPLC. From the results summarized in Table 2, it is clear that ORF1188 protein hydrolyzed fucose-containing biantennary oligosaccharides specifically. In addition, we found that the enzyme exhibited higher activity for the substrate containing terminal sialic acid with the α2,3-linkage and that terminal fucosylation was not accepted as a substrate, at least using PA-terminal fucosyl asialotriantennary we examined.

**Table 2:** Relative hydrolase activity of recombinant proteins.

PA-oligosaccharide	Structure	Relative activity(%)^a^		
		1188	Beauveria	Cordyceps
PA-trimannosyl core		ND^b^	ND^b^	ND^b^
PA-oligomannoside(M5)		ND^b^	ND^b^	ND^b^
PA-oligomannoside(M6)		ND^b^	NDb	ND^b^
PA-oligomannoside(M8)		ND^b^	ND^b^	ND^b^
PA-oligomannoside(M9)		ND^b^	ND^b^	ND^b^
PA-agalactobiantennary		ND^b^	ND^b^	ND^b^
PA-asialobiantennary		ND^b^	ND^b^	ND^b^
PA-sialobiantennary		ND^b^	ND^b^	ND^b^
PA-asialotriantennary		ND^b^	ND^b^	1.3
PA-asialotetraantennary		ND^b^	ND^b^	ND^b^
PA-fucosyl trimannosyl core		ND^b^	ND^b^	ND^b^
PA-fucosyl agalactobiantennary		11.3	10.2	21.4
PA-fucosyl asialobiantennary		17.9	6.9	36.7
PA-fucosyl sialobiantennary		100	100	100
PA-fucosyl asialotriantennary		ND^b^	ND^b^	ND^b^
PA-terminal fucosyl asialotriantennary		ND^b^	ND^b^	ND^b^
Mannose GlcNAc Galactose Sialic acid Fucose				

^a^The relative activity value of each enzyme for a given PA-oligosaccharide was calculated with respect to its value for PA-fucosyl sialobiantennary, which was set at 100.

^b^ND, not detectable.

### Hydrolytic activities of recombinant ENGase against glycoproteins

**Figure 3:**
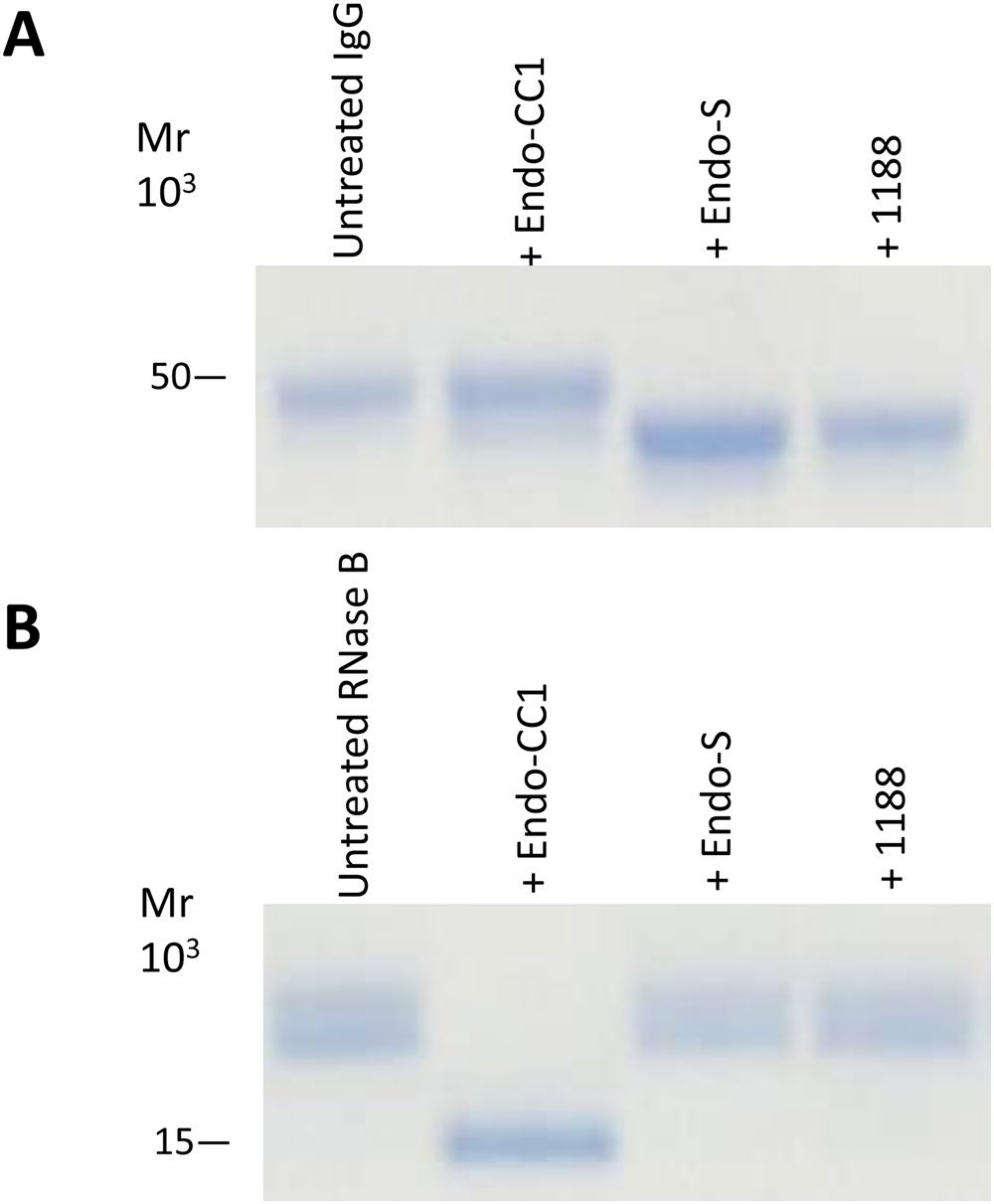
SDS-PAGE analysis of the hydrolytic activities of recombinant ORF1188 protein against rituximab and RNase B. Rituximab (**A**) or RNase B (**B**) was incubated separately with Endo-S, Endo-CC1, ORF1188 proteins overnight. Reaction mixtures were then subjected to SDS-PAGE analysis using either a 12% (for rituximab) or a 15% (for RNase B) acrylamide gel. Untreated rituximab (IgG) and RNase B were used as negative controls in (**A**) and (**B**), respectively. After SDS-PAGE, gels were stained with CBB. In (**A**), the heavy chain of IgG migrated as at approximately 50 kDa protein.

Next, we determined whether these recombinant protein could hydrolyze the *N*-linked glycans of glycoproteins. We selected RNase B and rituximab as glycoproteins containing high-mannose and fucosyl sialobiantennary type oligosaccharides, respectively. First, by using HPLC we confirmed that most of the oligosaccharide structure on rituximab was indeed fucosyl sialobiantennary (Fig. S2). We then found that the ORF1188 recombinant protein was able to hydrolyze rituximab (Fig. 3A), but not RNase B (Fig. 3B), suggesting that the recombinant protein ORF1188 can hydrolytically remove fucose-containing oligosaccharides from glycoproteins.

### ORF1188 ENGase homologs and their enzymatic characterization

**Figure 4:**
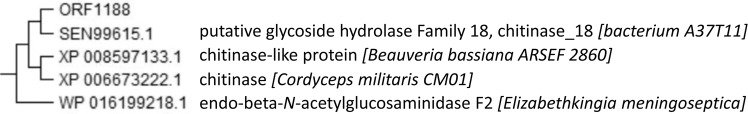
Phylogenetic tree of *Sphingobacterium* ENGase homologs. The amino acid sequences of hydrolases belonging to the GH18 family were retrieved by BLAST searches using the sequences of ORF1188 ENGase and these retrieved sequences were analyzed using the program CLUSTAL W to obtain the phylogenetic tree. The DDBJ accession numbers of sequences used for creating the phylogenetic tree are shown.

**Figure 5:**
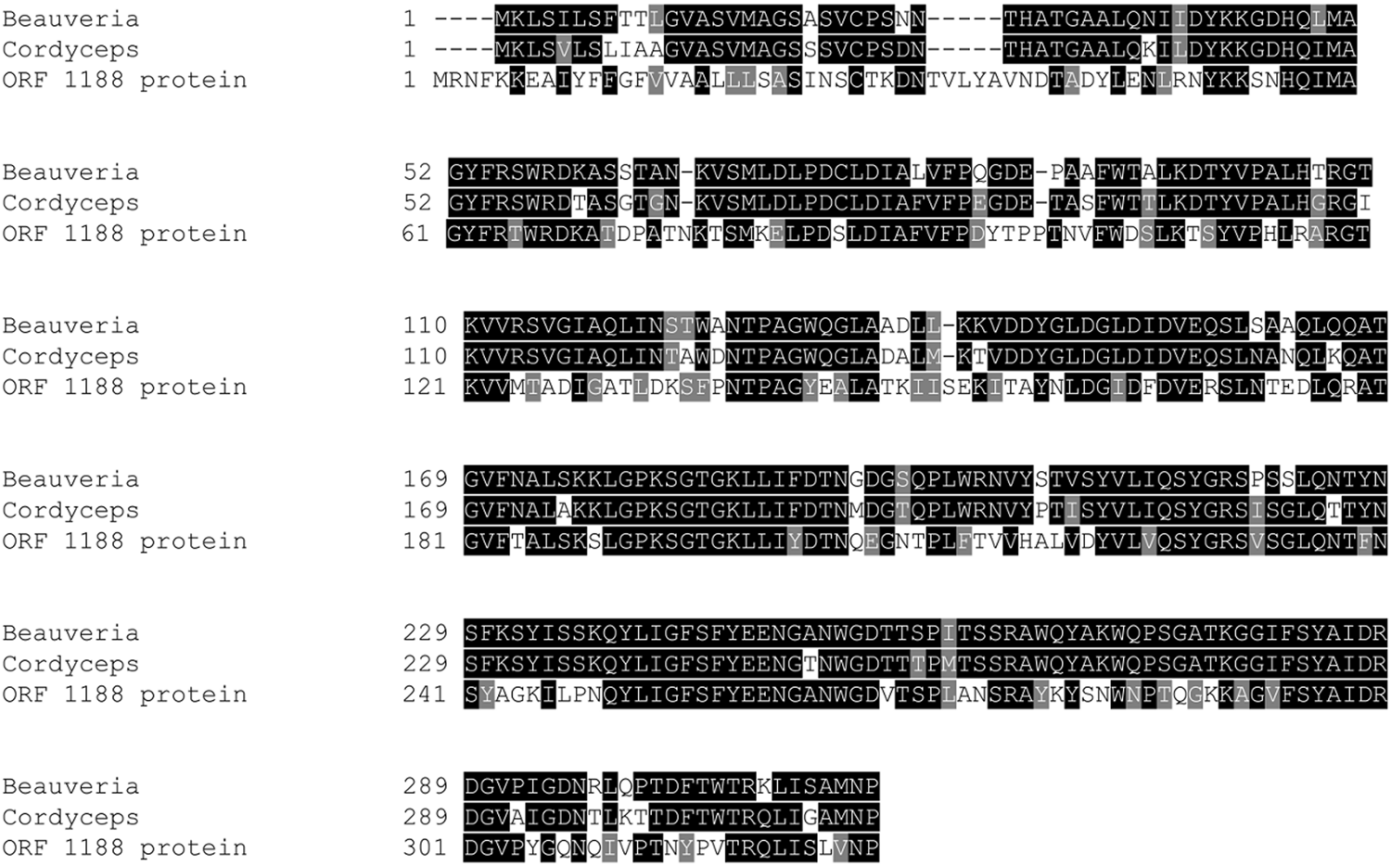
Sequence alignment of ORF1188, *Beauveria* and *Cordyceps* ENGases. Alignment of amino acid sequences of ORF1188, *Beauveria* and *Cordyceps* ENGases was shown. *Beauveria* and *Cordyceps* ENGases are consisted of 315 amino acid residues and they respectively show 54% and 53% sequence similarities to the ORF1188 ENGase.

**Figure 6:**
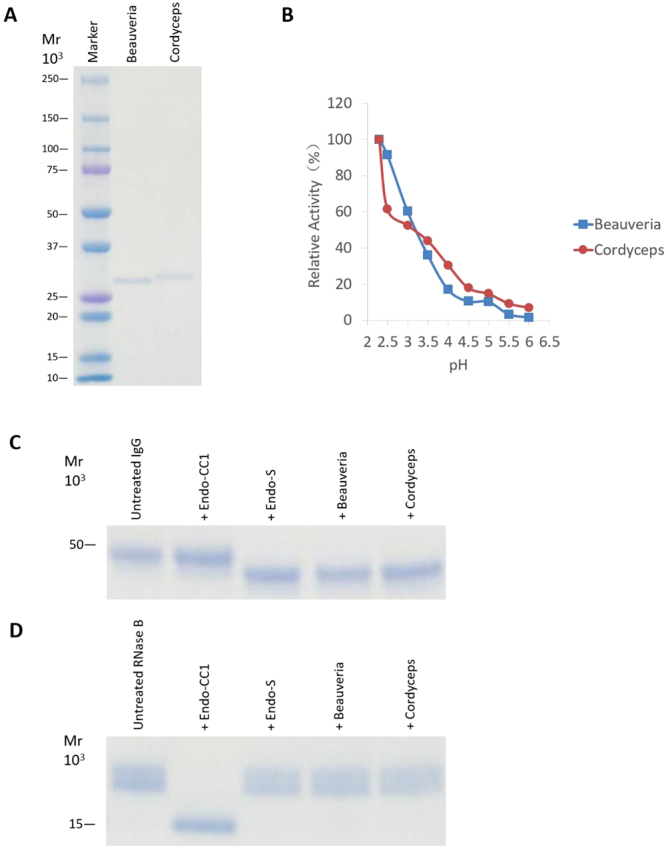
Characterization of *Beauveria* and *Cordyceps* ENGases. (**A**) Recombinant *Beauveria* and *Cordyceps* ENGases were expressed in *E. coli* and were purified. Protein samples (0.5 μg of each) were then subjected to SDS-PAGE using a 5–20% acrylamide gel, following which the gel was stained with CBB. (**B**) Effects of pH on the enzymatic activity of *Beauveria* and *Cordyceps* ENGases. The assay was carried out using 100 mM buffers at various pHs. (**C**) Rituximab (IgG) was incubated separately with Endo-S, Endo-CC1, recombinant *Beauveria* ENGase and recombinant *Cordyceps* ENGase overnight and subsequently analyzed by SDS-PAGE. The CBB stained gel shows the heavy chain of IgG is migrating as a protein with MW of approximately 50 kDa. (**D**) RNase B was incubated separately with Endo-S, Endo-CC1, recombinant *Beauveria* ENGase and recombinant *Cordyceps* ENGase overnight and subsequently analyzed by SDS-PAGE.

**Figure 7:**
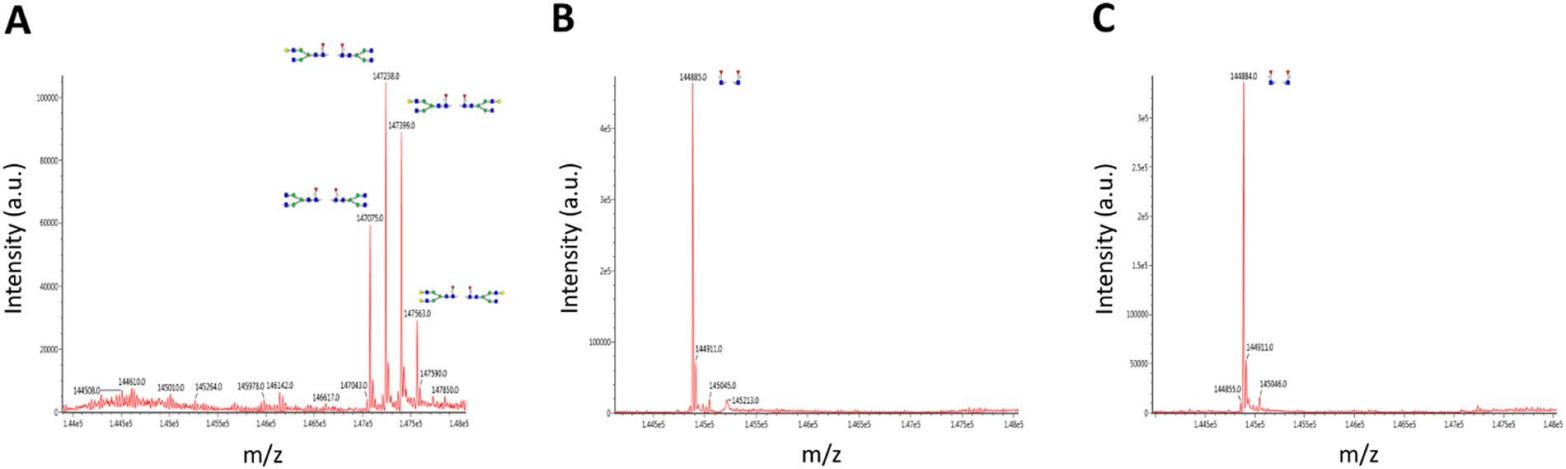
Confirmation of oligosaccharide structure on rituximab after ENGase treatment. LC-MS analysis was performed on the following samples: (**A**) rituximab without ENGase treatment (**B**) treated with ORF1188 and (**C**) treated with *Cordyceps* ENGase. Note that the single peak derived from GlcNAc-fucose was observed in samples (**B**) and (**C**), but not in sample (**A**).

Next, we performed BLAST searches using the sequence of ORF1188 ENGase from *Sphingobacterium* sp. and found homologs of the protein (Fig. 4). Among them, we selected two fungal homologs, one in *B. bassiana* and the other in *C. militaris*, both of which exhibited high degree of amino acid sequence similarity to ORF1188 (Fig. 5), for further characterization. For this purpose, we cloned genes encoding these homologs and expressed these genes in *E. coli* to obtain respective recombinant proteins. We then found that they specifically cleaved fucose-containing synthetic oligosaccharide substrates (Table 2) and also cleaved fucose-containing biantennary complex type oligosaccharides on rituximab (IgG), but did not cleave the high-mannose type oligosaccharides on RNase B (Fig. 6). Finally, we treated rituximab either with ORF1188 ENGase or *Cordyceps* ENGase and then analyzed the oligosaccharide structure of the treated rituximab using LC-MS; in both cases, we observed a signal derived from GlcNAc-fucose (Fig. 7), confirming that both ENGases hydrolyzed fucose-containing biantennary complex type oligosaccharides.

## Discussion

In this study, we identified three novel ENGases, one from *Sphingobacterium* sp., one from *B. bassiana* and one from *C. militaris*. All of these enzymes can catalyze the hydrolysis of fucose-containing biantennary complex type oligosaccharides, but not that of high-mannose type oligosaccharides, a characteristic feature that is highly unique compared to other previously characterized ENGases. Each one of these novel ENGases consisted of around 300 amino acid residues, a number that is much smaller than the Endo-S that consists of about 1,000 amino acid residues. Their smaller sizes enabled us to easily purify them as recombinant proteins from *E. coli* cultures. One possible disadvantage of using these ENGases for biomedical applications, such as in releasing oligosaccharides off IgG, is that they are optimally active at a quite acidic pH. However, ORF1188 ENGase exhibited about 40% remaining activity at pH 5, thus suggesting that these novel ENGases could be used for evaluating oligosaccharides on pharmaceutical glycoproteins, including IgG.

Although protein glycosylation is the most widespread and diverse post-translational modification that profoundly affects protein function^16^, yet glycosylated proteins exhibit structural micro-heterogeneity in the oligosaccharide moiety. Therefore, there is an urgent need for the synthesis of homogeneous glycoproteins with well-defined glycans for structure-function relationship studies and also for biomedical applications. Recently, a novel chemoenzymatic glycosylation engineering technology, which involves ENGase-catalyzed deglycosylation followed by transglycosylation of intact glycoproteins, was developed as a promising strategy to elegantly achieve glycan-defined glycoproteins^17^. Indeed, several therapeutic antibodies with homogeneously modified glycan structure, created using this strategy, exhibited enhanced antibody-dependent cellular cytotoxicity (ADCC) and complement-dependent cytotoxicity (CDC)^18,19^. Several glycosynthases were also successfully created by site-directed mutagenesis from different endoglycosidases, which include Endo-A^20,21^, Endo-M^22^, Endo-D^23^ and Endo-CC1^15^ of GH85 family as well as Endo-S^24,25^, Endo-F3^26^ and Endo-S2^27^ of GH18 family. However, it is not known yet whether Endo-SBs possibly have transglycosylation activity. We are currently investigating the transglycosylation activities of Endo-SBs and their mutants.

It has been demonstrated that the conserved *N*-linked glycan of IgG, which contains fucosylated oligosaccharides, are of significant importance in antibody-mediated immune responses, such as in ADCC and CDC^28–31^. Therefore, enzymes that can act on fucosylated *N*-glycans could have potential application as a therapeutic agent for the treatment of antibody-induced autoimmune diseases. Consistent with this notion, Endo-S has been successfully developed as a therapy to inhibit various experimental autoimmune disorders in many animal models^32^, including collagen-induced arthritis^33^, lethal IgG-driven immune thrombocytopenic purpura^34^, pathology in lupus-prone mice^35^, anti-neutrophil cytoplasmic autoantibodies (ANCA)-mediated glomerulonephritis^36^, antibody-mediated red blood cell (RBC) destruction^37^, systemic lupus erythematosus (SLE)^38^, autoimmunity to type VII collagen^39^ and myelin oligodendrocyte glycoprotein peptide amino acid 35–55 (MOG35-55)-induced experimental autoimmune encephalomyelitis (EAE)^40^. Since the catalytic activities of Endo-SBs are similar to the catalytic activity of Endo-S in hydrolytically cleaving the oligosaccharides on rituximab, potentially Endo-SBs could also be used as a therapeutic agent.

In summary, ENGases identified in this report could be useful as tools not only for analyzing the oligosaccharide contents of antibodies (basic research), but they could also be useful as tools for developing therapeutic antibody pharmaceuticals (biomedical application), such as an immunomodulatory therapeutic agent for the treatment of autoimmune disorders. In a recent study, it was reported that W251N mutant of Endo-M can catalyze the hydrolysis of fucosylated *N*-glycans, a feature is not seen in wild-type Endo-M^41^. At present, we do not know which amino acid residues in Endo-SBs are specifically involved in recognizing fucosylated oligosaccharides, because Endo-SBs belong to the GH18 family, whereas the Endo-M belongs to the GH85 family. We are currently in the process of analyzing the crystal structures of Endo-SBs in order to elucidate the underlying reason why Endo-SBs catalyze fucosylated *N*-glycans specifically.

## Electronic supplementary material

Supplementary information
