# Author Correction: Characterization of novel endo-β-*N*-acetylglucosaminidases from *Sphingobacterium* species, *Beauveria bassiana* and *Cordyceps militaris* that specifically hydrolyze fucose-containing oligosaccharides and human IgG

**DOI:** 10.1038/s41598-020-63704-2

**Published:** 2020-04-29

**Authors:** Yibo Huang, Yujiro Higuchi, Takashi Kinoshita, Ai Mitani, Yasunari Eshima, Kaoru Takegawa

**Affiliations:** 1 Department of Bioscience and Biotechnology, Faculty of Agriculture, Kyushu University, 6-10-1 Hakozaki, Fukuoka, 812-8581 Japan; 2 Fushimi Pharmaceutical Co. Ltd., Marugame, Kagawa 763-8605 Japan

Correction to: *Scientific Reports* 10.1038/s41598-017-17467-y, published online 10 January 2018

In the original version of the Article, we reported that ORF1152, ORF3046 and ORF3750 exhibited fucose-containing oligosaccharides-specific ENGase activity. However, recent re-experiments have shown that these proteins have no such activity. This error was caused by contamination with ORF1188, which indeed exhibits fucose-containing oligosaccharides-specific ENGase activity, in the protein purification procedures. We also confirmed that *Beuveria* and *Cordyceps* ENGases have fucose-containing oligosaccharides-specific ENGase activity like ORF1188.

The Article has thus been amended as follows: (1) Data regarding ORF1152, ORF3046 and ORF3750 have been removed in Table 2, Fig. 2, Fig. 3, Fig. 4 and Supplementary Fig. 1. (2) Other text referring to ORF1152, ORF3046 and ORF3750 has been deleted.

As a result, in the Abstract,

“Recombinant proteins, purified from *Escherichia coli* expressing candidate genes ORF1152, ORF1188, ORF3046 and ORF3750 exhibited fucose-containing oligosaccharides-specific ENGase activity. These ENGases exhibited optimum activities at very acidic pHs (between pH 2.3–2.5). BLAST searches using sequences of these candidate genes identified two fungal homologs of ORF1188, one in *Beauveria bassiana* and the other in *Cordyceps militaris*.”

now reads:

“Among them, a recombinant protein purified from *Escherichia coli* expressing the candidate gene ORF1188 exhibited fucose-containing oligosaccharides-specific ENGase activity. The ENGase exhibited optimum activities at very acidic pHs (between pH 2.3 – 2.5). A BLAST search using the sequence of ORF1188 identified two fungal homologs, one in *Beauveria bassiana* and the other in *Cordyceps militaris*.”

In the Materials and Methods, subheading ‘Analysis of hydrolase activity of ENGase proteins’,

“For this assay, since PA-sialobiantennary with terminal fucose was not commercially available, either 2 pmol of PA-sialobiantennary with core fucose (PA-fucosyl sialobiantennary) was mixed with a given amount of one of the newly identified *Sphingobacterium* ENGase recombinant proteins (3.4 ng of ORF1188, 11 ng of ORF1152, 220 ng of ORF3046 or 55 ng of ORF3750) or 2 pmol of PA-sialobiantennary was mixed either with 40 ng of recombinant *Beauveria* ENGase or with 77 ng of recombinant *Cordyceps* ENGase.”

now reads:

“For this assay, since PA-sialobiantennary with terminal fucose was not commercially available, either 2 pmol of PA-sialobiantennary with core fucose (PA-fucosyl sialobiantennary) was mixed with a given amount of one of the newly identified *Sphingobacterium* ENGase recombinant protein (3.4 ng of ORF1188) or 2 pmol of PA-sialobiantennary was mixed either with 40 ng of recombinant *Beauveria* ENGase or with 77 ng of recombinant *Cordyceps* ENGase.”

And,

“To determine the substrate specificities of these ENGases, 2 pmol of each PA-labeled-oligosaccharide (Takara and Masuda Chemical) was mixed with a given amount of one of the recombinant ENGases (1.2 ng of ORF1188, 54 ng of ORF1152, 42 ng of ORF3046, 124 ng of ORF3750, 1 ng of *Beauveria* or 1.2 ng of *Cordyceps*) in 10 μL of 100 mM acetate buffer (pH 3) and the mixture was incubated at 30 °C for 20 min, following which the reaction was stopped by incubating the mixture at 99 °C for 10 min.”

now reads:

“To determine the substrate specificities of these ENGases, 2 pmol of each PA-labeled-oligosaccharide (Takara and Masuda Chemical) was mixed with a given amount of one of the recombinant ENGases (1.2 ng of ORF1188, 1 ng of *Beauveria* or 1.2 ng of *Cordyceps*) in 10 μL of 100 mM acetate buffer (pH 3) and the mixture was incubated at 30°C for 20 min, following which the reaction was stopped by incubating the mixture at 99°C for 10 min.”

Furthermore, in the same subheading,

“To determine whether these recombinant ENGase proteins could hydrolyze glycoproteins, a given amount of each recombinant protein (ORF1188, ORF3046, ORF3750, 5 μg each; 3 μg of ORF1152; *Beauveria* and *Cordyceps* ENGases, 1 μg each) in 50 μL of 100 mM acetate buffer (pH 5.0) was incubated either with 10 μg of RNase B (Sigma) or with 10 μg of rituximab (Rituxan®; Zenyaku Kogyo Co., Ltd.) overnight at 30 °C.”

now reads:

“To determine whether these recombinant ENGase proteins could hydrolyze glycoproteins, a given amount of each recombinant protein (5 μg of ORF1188; *Beauveria* and *Cordyceps* ENGases, 1 μg each) in 50 μL of 100 mM acetate buffer (pH 5.0) was incubated either with 10 μg of RNase B (Sigma) or with 10 μg of rituximab (Rituxan®; Zenyaku Kogyo Co., Ltd.) overnight at 30°C.”

In the Results, subheading ‘Characterization of hydrolase activity of recombinant proteins’,

“To characterize the hydrolase activity of the putative *Sphingobacterium* ENGases, we individually expressed ORF1152 (without signal peptide), ORF1188 (without signal peptide), ORF3046 and ORF3750 in *E. coli*, and successfully purified these recombinant proteins from the respective *E. coli* cultures grown at 15 °C, as judged by SDS-PAGE analysis (Fig. 2A; images of original gels are shown in Fig. S1). Using these purified proteins, we determined that the hydrolase activities of both ORF1188 and ORF3046 proteins were optimum at pH 2.5 and the hydrolase activities of both ORF1152 and ORF3750 proteins were optimum at pH 2.3 (Fig. 2B). These pH values for optimal activity are much lower than those observed for other ENGases.

“Next, we characterized substrate specificities of ORF1152, ORF1188, ORF3046 and ORF3750 proteins. The relative activity of each protein was measured using varieties of PA-oligosaccharides as substrates, and the hydrolyzed products were analyzed by HPLC. From the results summarized in Table 2, it is clear that all four proteins hydrolyzed fucose-containing biantennary oligosaccharides specifically. In addition, we found that these four enzymes exhibited higher activity for the substrate containing terminal sialic acid with the α2,3-linkage and that terminal fucosylation was not accepted as a substrate, at least using PA-terminal fucosyl asialotriantennary we examined.”

now reads:

“To characterize the hydrolase activity of the putative *Sphingobacterium* ENGases, we individually expressed ORF1152 (without signal peptide), ORF1188 (without signal peptide), ORF3046 and ORF3750 in *E. coli*, and successfully purified these recombinant proteins from the respective *E. coli* cultures grown at 15°C, as judged by SDS-PAGE analysis (Fig. 2A; images of original gels are shown in Fig. S1). Among these purified proteins, we found that only ORF1188 protein exhibited the ENGase activity. We further determined that the hydrolase activity of ORF1188 protein was optimum at pH 2.5 (Fig. 2B). The pH value for optimal activity is much lower than those observed for other ENGases.

“Next, we characterized substrate specificities of ORF1188 protein. The relative activity of the protein was measured using varieties of PA-oligosaccharides as substrates, and the hydrolyzed products were analyzed by HPLC. From the results summarized in Table 2, it is clear that ORF1188 protein hydrolyzed fucose-containing biantennary oligosaccharides specifically. In addition, we found that the enzyme exhibited higher activity for the substrate containing terminal sialic acid with the α2,3-linkage and that terminal fucosylation was not accepted as a substrate, at least using PA-terminal fucosyl asialotriantennary we examined.”

Additionally, under the subheading ‘Hydrolytic activities of recombinant ENGases against glycoproteins’,

“Next, we determined whether these recombinant proteins could hydrolyze the *N*-linked glycans of glycoproteins. We selected RNase B and rituximab as glycoproteins containing high-mannose and fucosyl sialobiantennary type oligosaccharides, respectively. First, by using HPLC we confirmed that most of the oligosaccharide structure on rituximab was indeed fucosyl sialobiantennary (Fig. S2). We then found that all four recombinant proteins were able to hydrolyze rituximab (Fig. 3A), but not RNase B (Fig. 3B), suggesting that the recombinant proteins ORF1152, ORF1188, ORF3046 and ORF3750 can hydrolytically remove fucose-containing oligosaccharides from glycoproteins.”

now reads:

“Next, we determined whether these recombinant protein could hydrolyze the *N*-linked glycans of glycoproteins. We selected RNase B and rituximab as glycoproteins containing high-mannose and fucosyl sialobiantennary type oligosaccharides, respectively. First, by using HPLC we confirmed that most of the oligosaccharide structure on rituximab was indeed fucosyl sialobiantennary (Fig. S2). We then found that the ORF1188 recombinant protein was able to hydrolyze rituximab (Fig. 3A), but not RNase B (Fig. 3B), suggesting that the recombinant protein ORF1188 can hydrolytically remove fucose-containing oligosaccharides from glycoproteins.”

Furthermore, under the subheading ‘ORF1188 ENGase homologs and their enzymatic characterization’,

“Next, we performed BLAST searches using the sequences of five putative ENGases from *Sphingobacterium* sp. and found homologs of these proteins in a wide range of organisms, ranging from bacteria to eukaryotes (Fig. 4).”

now reads:

“Next, we performed BLAST searches using the sequence of ORF1188 ENGase from *Sphingobacterium* sp. and found homologs of the protein (Fig. 4).”

In the Discussion,

“In this study, we identified six novel ENGases, four from *Sphingobacterium* sp., one from *B. bassiana* and one from *C. militaris*.”

now reads:

“In this study, we identified three novel ENGases, one from *Sphingobacterium* sp., one from *B. bassiana* and one from *C. militaris*.”

In the legend of Figure 2,

“Analyses of molecular weights and pH-dependent activities of recombinant proteins. (**A**) ORF1188, ORF1152, ORF3046 and ORF3750 genes were expressed in *E. coli* as recombinant proteins and were purified. 0.5 μg of each purified protein sample was loaded onto a 15% acrylamide gel, and the gel was stained with CBB. (**B**) Effects of pH on the enzymatic activities of ORF1188, ORF1152, ORF3046 and ORF3750 proteins. Activity of each protein was assayed at various pHs using 100 mM acetate buffers.”

now reads:

“Analyses of molecular weight and pH-dependent activities of recombinant ORF1188 protein. (**A**) ORF1188 gene was expressed in *E. coli* as a recombinant protein and was purified. 0.5 μg of the purified protein sample was loaded onto a 15% acrylamide gel, and the gel was stained with CBB. (**B**) Effects of pH on the enzymatic activities of ORF1188 protein. Activity of the protein was assayed at various pHs using 100 mM acetate buffers.”

In the legend of Figure 3,

“SDS-PAGE analysis of the hydrolytic activities of recombinant proteins against rituximab and RNase B. Rituximab (**A**) or RNase B (**B**) was incubated separately with Endo-S, Endo-CC1, ORF1188, ORF1152, ORF3046 or ORF3750 proteins overnight.”

now reads:

“SDS-PAGE analysis of the hydrolytic activities of recombinant ORF1188 protein against rituximab and RNase B. Rituximab (**A**) or RNase B (**B**) was incubated separately with Endo-S, Endo-CC1, ORF1188 proteins overnight.”

The legend of Figure 4,

“Phylogenetic tree of putative *Sphingobacterium* ENGase homologs. The amino acid sequences of hydrolases belonging to the GH18 family were retrieved by BLAST searches using the sequences of five candidate ENGases and these retrieved sequences were analyzed using the program CLUSTAL W to obtain the phylogenetic tree. The DDBJ accession numbers of sequences used for creating the phylogenetic tree are shown. Note that ORF1188 homologs exist not only in bacteria but also in fungi.”

now reads:

“Phylogenetic tree of *Sphingobacterium* ENGase homologs. The amino acid sequences of hydrolases belonging to the GH18 family were retrieved by BLAST searches using the sequences of ORF1188 ENGase and these retrieved sequences were analyzed using the program CLUSTAL W to obtain the phylogenetic tree. The DDBJ accession numbers of sequences used for creating the phylogenetic tree are shown.”

This has now been corrected in the HTML and PDF versions of this Article, and in the accompanying Supplemental Material.

The original version of Table 2 is included below as Table 1.

**Table 1:** Relative hydrolase activity of recombinant proteins.

PA-oligosaccharide	Structure	Relative activity(%)^a^					
		1188	1152	3046	3750	Beauveria	Cordyceps
PA-trimannosyl core		ND^b^	ND^b^	ND^b^	ND^b^	ND^b^	ND^b^
PA-oligomannoside(M5)		ND^b^	ND^b^	ND^b^	ND^b^	ND^b^	ND^b^
PA-oligomannoside(M6)		ND^b^	ND^b^	ND^b^	0.5	NDb	ND^b^
PA-oligomannoside(M8)		ND^b^	ND^b^	ND^b^	ND^b^	ND^b^	ND^b^
PA-oligomannoside(M9)		ND^b^	ND^b^	ND^b^	ND^b^	ND^b^	ND^b^
PA-agalactobiantennary		ND^b^	ND^b^	ND^b^	ND^b^	ND^b^	ND^b^
PA-asialobiantennary		ND^b^	ND^b^	ND^b^	ND^b^	ND^b^	ND^b^
PA-sialobiantennary		ND^b^	ND^b^	ND^b^	0.7	ND^b^	ND^b^
PA-asialotriantennary		ND^b^	ND^b^	ND^b^	ND^b^	ND^b^	1.3
PA-asialotetraantennary		ND^b^	ND^b^	ND^b^	ND^b^	ND^b^	ND^b^
PA-fucosyl trimannosyl core		ND^b^	ND^b^	ND^b^	1.7	ND^b^	ND^b^
PA-fucosyl agalactobiantennary		11.3	11.8	8.3	8.2	10.2	21.4
PA-fucosyl asialobiantennary		17.9	41.1	25.6	49.9	6.9	36.7
PA-fucosyl sialobiantennary		100	100	100	100	100	100
PA-fucosyl asialotriantennary		ND^b^	ND^b^	ND^b^	ND^b^	ND^b^	ND^b^
PA-terminal fucosyl asialotriantennary		ND^b^	ND^b^	ND^b^	ND^b^	ND^b^	ND^b^
Mannose GlcNAc Galactose Sialic acid Fucose							

^a^The relative activity value of each enzyme for a given PA-oligosaccharide was calculated with respect to its value for PA-fucosyl sialobiantennary, which was set at 100.

^b^ND, not detectable.

The original versions of Figures 2, 3 and 4 are included below as Figures 1, 2 and 3.

**Figure 1:**
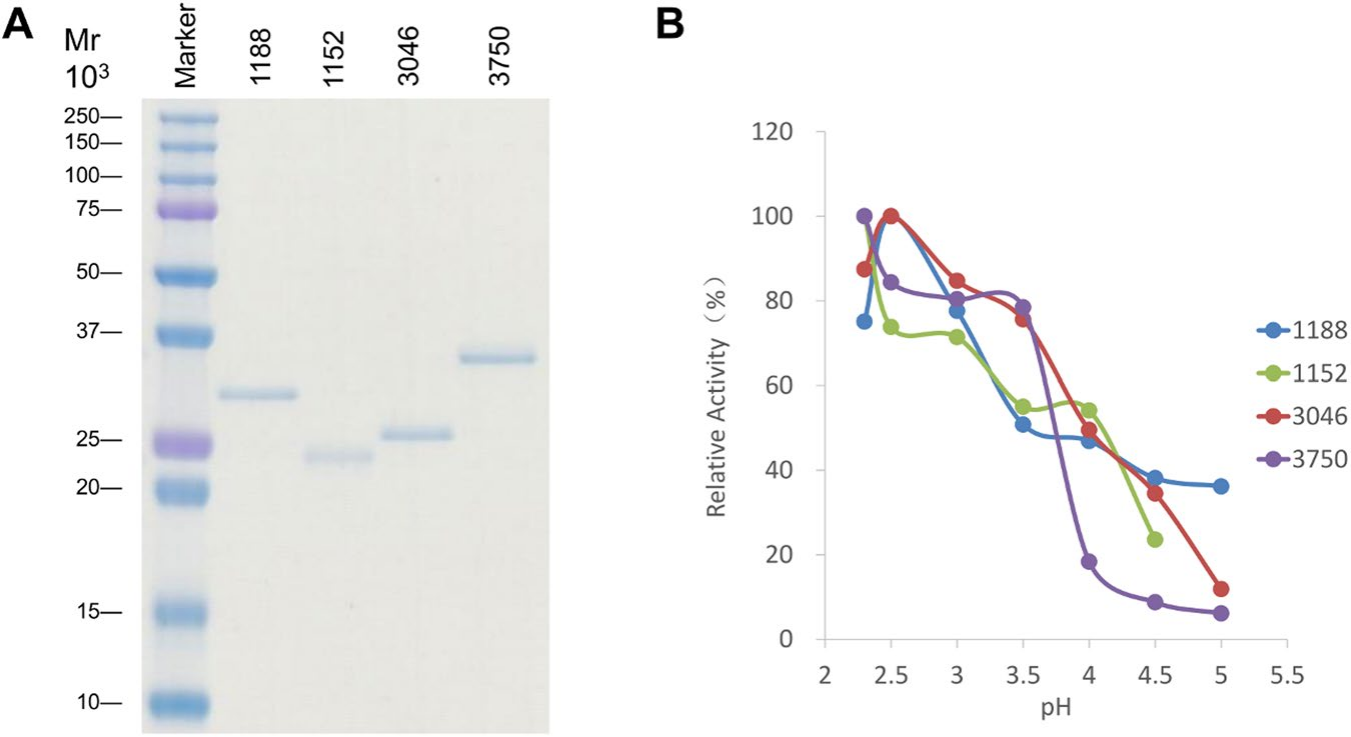
Analyses of molecular weights and pH-dependent activities of recombinant proteins. (**A**) ORF1188, ORF1152, ORF3046 and ORF3750 genes were expressed in *E. coli* as recombinant proteins and were purified. 0.5 μg of each purified protein sample was loaded onto a 15% acrylamide gel, and the gel was stained with CBB. (**B**) Effects of pH on the enzymatic activities of ORF1188, ORF1152, ORF3046 and ORF3750 proteins. Activity of each protein was assayed at various pHs using 100 mM acetate buffers.

**Figure 2:**
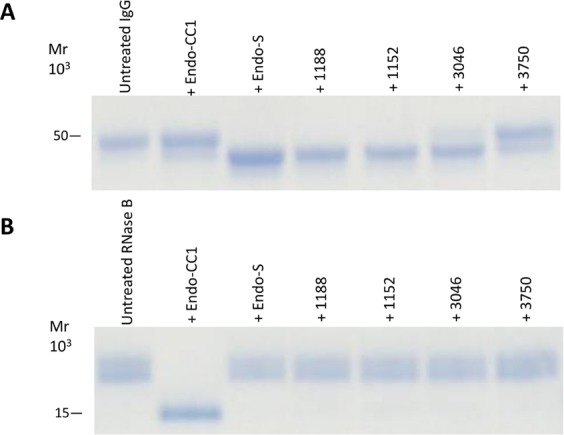
SDS-PAGE analysis of the hydrolytic activities of recombinant proteins against rituximab and RNase B. Rituximab (**A**) or RNase B (**B**) was incubated separately with Endo-S, Endo-CC1, ORF1188, ORF1152, ORF3046 or ORF3750 proteins overnight. Reaction mixtures were then subjected to SDS-PAGE analysis using either a 12% (for rituximab) or a 15% (for RNase B) acrylamide gel. Untreated rituximab (IgG) and RNase B were used as negative controls in (**A**) and (**B**), respectively. After SDS-PAGE, gels were stained with CBB. In (**A**), the heavy chain of IgG migrated as at approximately 50 kDa protein.

**Figure 3:**
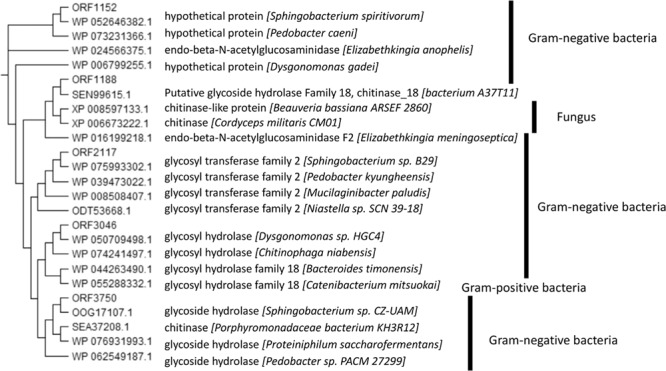
Phylogenetic tree of putative *Sphingobacterium* ENGase homologs. The amino acid sequences of hydrolases belonging to the GH18 family were retrieved by BLAST searches using the sequences of five candidate ENGases and these retrieved sequences were analyzed using the program CLUSTAL W to obtain the phylogenetic tree. The DDBJ accession numbers of sequences used for creating the phylogenetic tree are shown. Note that ORF1188 homologs exist not only in bacteria but also in fungi.

The Article’s original Supplementary Information file has been included here.

## Supplementary information

Supplementary information

