# Supplementary material for: Author Correction: Characterization of novel endo-β-N-acetylglucosaminidases from Sphingobacterium species, Beauveria bassiana and Cordyceps militaris that specifically hydrolyze fucose-containing oligosaccharides and human IgG
Source: Sci Rep. 2020 Apr 29;10:7413. doi: 10.1038/s41598-020-63704-2 (PMC7190819; doi:10.1038/s41598-020-63704-2)
Supplement: Supplementary file 1 — Supplementary information [file 41598_2020_63704_MOESM1_ESM.pdf]

Supplementary Information

Characterization of novel endo-β-*N*-acetylglucosaminidases from *Sphingobacterium* species, *Beauveria bassiana* and *Cordyceps militaris* that specifically hydrolyze fucose-containing oligosaccharides and human IgG

Yibo Huang[1], Yujiro Higuchi[1], Takashi Kinoshita[2], Ai Mitani[2], Yasunari Eshima[1], Kaoru Takegawa[1]

[1]Department of Bioscience and Biotechnology, Faculty of Agriculture, Kyushu University, 6-10-1 Hakozaki, Fukuoka 812-8581, Japan

[2]Fushimi Pharmaceutical Co. Ltd., Marugame, Kagawa 763-8605, Japan

[*]Corresponding author. Tel/Fax: +81 92 642 2851, E-mail address: takegawa@agr.kyushu-u.ac.jp (K. Takegawa)

Word count: Abstract, 193; Main text, 3259.

Number of figures: Main, 7; Supplementary, 2.

Number of tables: Main, 2; Supplementary, 1.

Running title: Characterization of *Sphingobacterium* endo-β-*N*-acetylglucosaminidases.

Supplementary figure legends

Supplementary figure 1. Images of original SDS-PAGE gels cropped and used in main figures.

Images of the following original SDS-PAGE gels were cropped and used in the figures as follows: (A) in Figure 3A; (B) in Figure 3B; (C) in Figure 6C; and (D) in Figure 6D.

Supplementary figure 2. Analysis of oligosaccharide structure on rituximab.

HPLC analysis was performed to determine oligosaccharide structures on rituximab. Note that most of the oligosaccharide structures are fucosyl sialobiantennary.

Supplementary Fig. 1 Huang et al

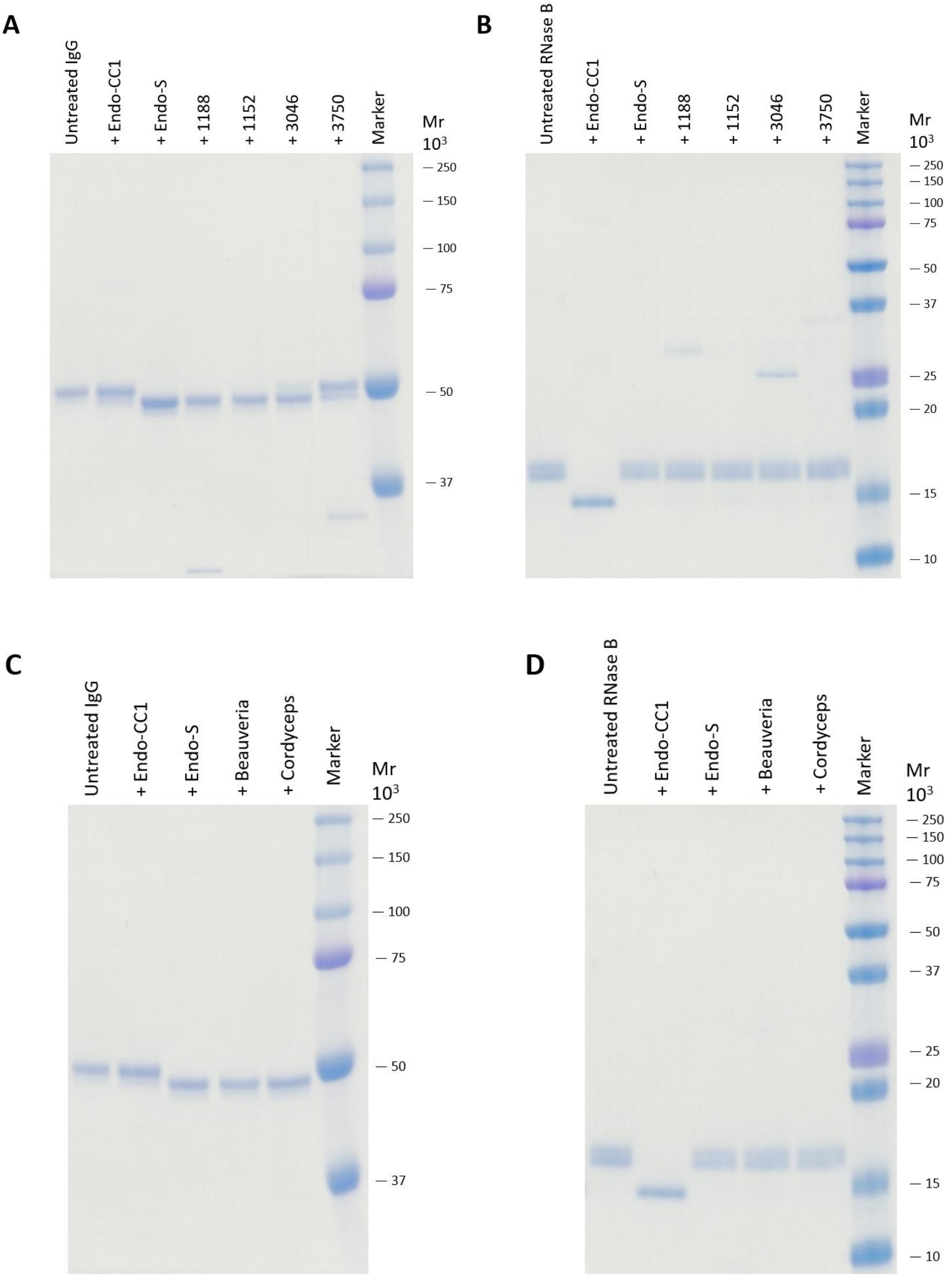

Supplementary Fig. 2 Huang et al

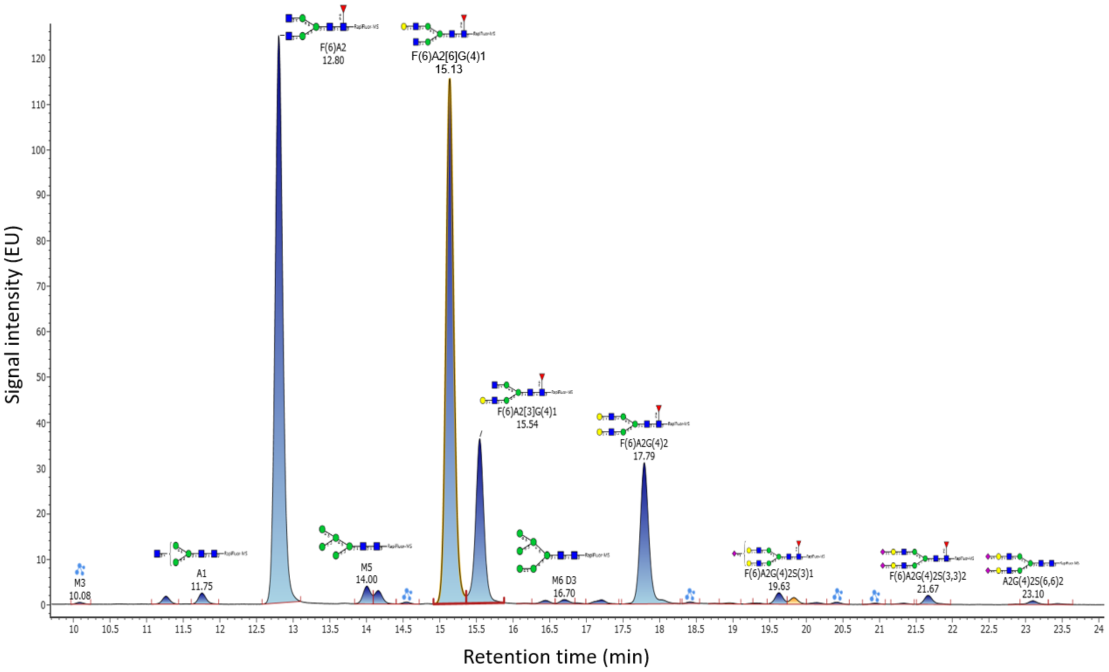

Supplementary Table 1 Primers used in this study

Target	Sequence (5' to 3'; upper, forward; lower, reverse)
pET-32b	ATGTATATCTCCTTCTTAAAGTTAAAC
	CTCGAGCACCACCACCACCACCACTGAG
ORF1188	GAAGGAGATATACATATGAAAGATAACACCGTGCTGTATG
	GTGGTGGTGCTCGAGTGGATTGACCAATGAAATCAAC
ORF1152	GAAGGAGATATACATATGGCCTGCGAAAAGCAGAATATTC
	GTGGTGGTGCTCGAGACGTACAGCGGGATTCATAATC
ORF3046	GAAGGAGATATACATATGTTCCATAAAAAATTAATAACC
	GTGGTGGTGCTCGAGCTTTCCTATGGACCAGTGGATAGC
ORF3750	GAAGGAGATATACATATGAAATCCAACCATCTTACAAAT
	GTGGTGGTGCTCGAGACGTATAATACCCTTATACACCGC
Beauveria	GAAGGAGATATACATATGGGTAGCGCAAGCGTTTGTCC
	GTGGTGGTGCTCGAGCGGATTCATTGCGCTAATC
Cordyceps	GAAGGAGATATACATATGGGTAGCAGCAGCGTTTGTCC
	GTGGTGGTGCTCGAGCGGATTCATTGCACCGATC
